# Repeatability and prognostic value of radiomic features: a study in esophageal cancer and nasopharyngeal carcinoma

**DOI:** 10.1186/s13244-025-02044-z

**Published:** 2025-08-02

**Authors:** Jie Gong, Fan Meng, Changhao Liu, Jianchao Lu, Jie Li, Zhi Yang, Hongfei Sun, Xinzhi Teng, Jiang Zhang, Jing Cai, Mei Shi, Lina Zhao

**Affiliations:** 1 State Key Laboratory of Holistic Integrative Management of Gastrointestinal Cancers and Department of Radiation Oncology, Xijing Hospital, Fourth Military Medical University, Xi’an, China; 2 Department of Radiation Oncology, Sichuan Cancer Hospital and Institution, Sichuan Cancer Center, School of Medicine, University of Electronic Science and Technology of China, Chengdu, China; 3 Department of Health Technology and Informatics, The Hong Kong Polytechnic University, Hung Hom, Hong Kong SAR

**Keywords:** Radiomics, Repeatability, Prognosis, Esophageal cancer, Nasopharyngeal carcinoma

## Abstract

**Objectives:**

To investigate whether radiomic features (RFs) repeatability and their prognostic value are study-specific.

**Materials and methods:**

This retrospective study included 234 esophageal cancer (EC) patients (contrast-enhanced computed tomography (CECT) and fluorine-18 fluorodeoxyglucose positron emission tomography (PET)), and 525 nasopharyngeal carcinoma (NPC) patients (CECT). Tumor, peritumor, and lymph node regions were defined as regions of interest. RF repeatability was assessed via perturbation analysis using intraclass correlation coefficients (ICC), with consistency and differences across cancer types, pathological regions, and modalities evaluated. The independent prognostic features common to both EC and NPC were screened from highly repeatable features using *C*-index and redundancy analysis.

**Results:**

CT-based RFs in NPC and PET-based RFs in EC demonstrated significantly higher repeatability compared to CT-based RFs in EC (median ICC: 0.886 vs 0.806; 0.897 vs 0.806; *p* < 0.05). While CT-based peritumoral features showed comparable repeatability to tumor features in EC (0.824 vs 0.806, *p* > 0.05), PET-based peritumoral features exhibited significantly lower repeatability than tumor features (0.819 vs 0.897, *p* < 0.05). CT-based lymph node features demonstrated significantly lower repeatability than tumor features in NPC (0.863 vs 0.886, *p* < 0.05). Nevertheless, the effects of bin count, feature class, and filter on repeatability demonstrated consistent patterns across different cancer types, imaging modalities, and pathological regions. Moreover, four common independent prognostic features effectively stratified both EC and NPC patients into high- and low-risk groups with significant survival differences (*p* < 0.05).

**Conclusions:**

RF repeatability might be affected by cancer type, pathological region, and imaging modality, while certain features maintain consistent prognostic performance across different cancer types.

**Critical relevance statement:**

The identification of high-repeatable pan-cancer prognostic radiomics features enables noninvasive patient risk stratification to guide personalized therapy, with cross-cancer consistency enhancing their applicability and convenience in clinical practice, thereby accelerating the integration of radiomics into precision oncology clinical workflows.

**Key Points:**

- This study examined RF repeatability and prognostic value specificity.
- RF repeatability varies across cancer types, regions, and modalities.
- The common highly repeatable RFs advance pan-cancer biomarker precision oncology.

**Graphical Abstract:**

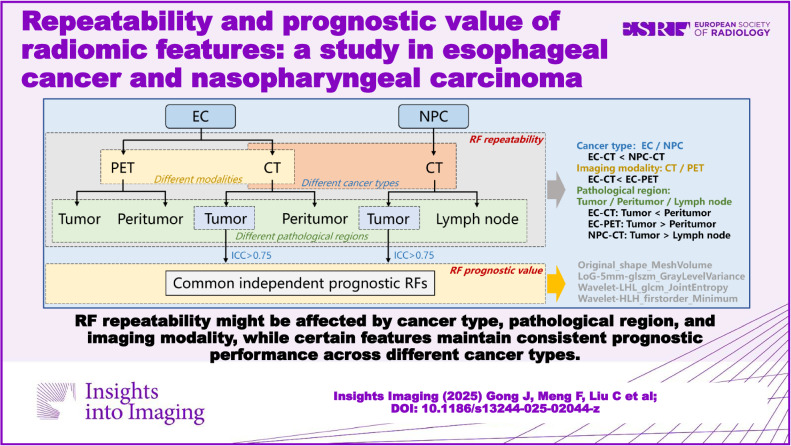

## Introduction

Radiomics, which extracts high-dimensional features from medical images to develop predictive models for disease diagnosis and prognosis, has emerged as a cornerstone of precision medicine [1–3]. The repeatability of radiomic feature (RF), defined as the ability to remain consistent when imaging the same subject under identical acquisition protocols [4], is critical to maintaining model stability and generalizability.

Current investigations of RF repeatability predominantly focus on specific malignancies, including nasopharyngeal carcinoma (NPC) [5], head-and-neck carcinoma [6], soft-tissue sarcomas [7], glioblastoma [8], cervical cancer [9], and lung cancer [10]. Whether an extensive study can identify a set of repeatable RFs that are universally applicable to radiomic analysis is unclear. Janna et al investigated and compared the RF repeatability of tumors in lung (*n* = 27) and rectal cancers (*n* = 40), demonstrating limited consistency [11]. More evidence is needed to prove whether the repeatability of RF is study-specific, not only in different disease types, but also in different pathological regions and different imaging modalities.

RFs provide quantitative descriptors of tumor pathophysiology [12], encoding morphological, textural, and intensity-based characteristics that surpass visual interpretation [2]. By harnessing machine learning and artificial intelligence algorithms, radiomics can decipher complex patterns, which in turn facilitate the prediction of tumor heterogeneity [1], aggressiveness [13], treatment response [14, 15], and even prognosis [16, 17]. This multiparametric approach augments our understanding of tumor biology by quantifying phenotypic variations that mirror genetic alterations, microenvironmental interactions, and metabolic activity, ultimately enriching our capacity to tailor precision medicine strategies and improve clinical outcomes. Further exploring the consistency of the prognostic ability of high-repeatable RFs in different cancer types has the potential to unravel the mysteries underlying both commonalities and distinctions in tumor biology.

Test–retest experiments, which assess feature consistency through short-interval scans, are a conventional evaluation method for RF repeatability, and have been utilized in several studies, including cervical cancer [9], lung cancer [10], and other diseases [18]. However, short-interval scans are uncommon in clinical practice, stemming from resource constraints and radiation exposure concerns, limiting extensive research on RF repeatability. Moreover, most of the relevant studies included limited samples, which reduced the stability and reliability of the results. To address these limitations, Zwanenburg et al proposed a perturbation-based RF robustness measurement alternative to test–retest imaging [19]. Zhang et al validated its effectiveness through direct comparison with test–retest approaches [4]. Subsequent applications in head-and-neck carcinoma [6] and NPC [5] have established perturbation analysis as a viable alternative.

In this study, we aimed to explore RF repeatability across different cancer types (esophageal cancer (EC)/NPC), pathological regions (tumor/peritumor/lymph nodes), and imaging modalities (CT/PET) using perturbation analysis, and to further evaluate the generalizability of the prognostic performance of high-repeatable RFs. Unlike previous studies that focused on single cancer types or imaging modalities, our work provides a comprehensive evaluation of RF repeatability, revealing for the first time the effects of cancer types, pathological regions, and imaging modalities on RF repeatability. This study could provide an important methodological reference for the study of radiomics, and also provide a new idea for the study of pan-cancer signatures.

## Methods

### Patients and images

**Fig. 1:**
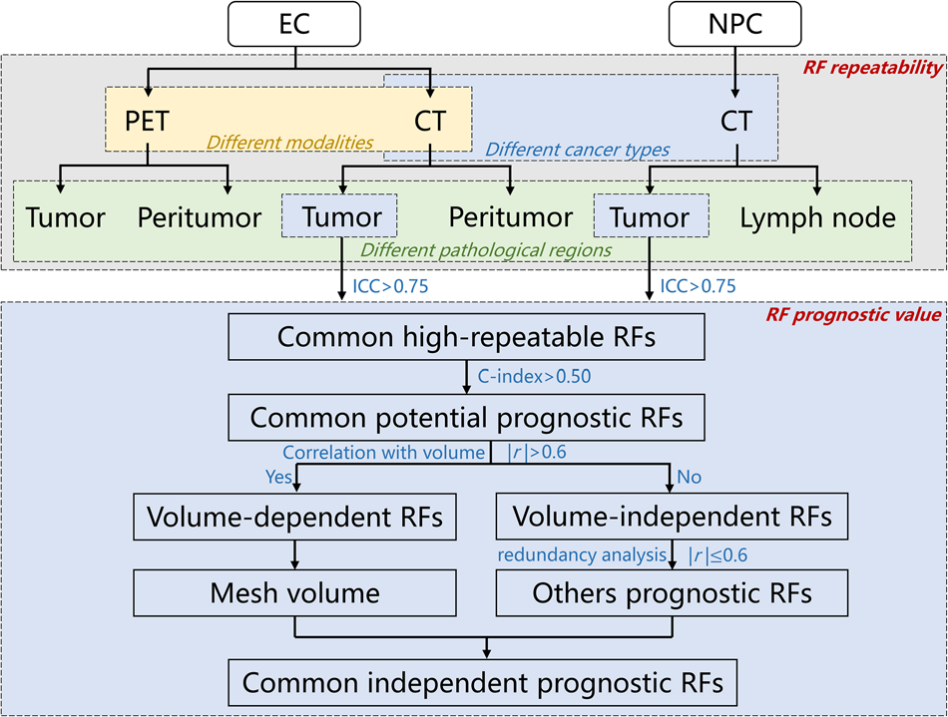
Flowchart of this study. The repeatability of RFs was assessed via image perturbation in different cancer types, different pathological regions, and different imaging modalities. The common independent prognostic features were gradually selected through the steps of feature repeatability, prognostic value, and redundancy analysis

This study was approved by the Xijing Hospital Ethics Committee (KY20222145-C-1), and the flowchart is shown in Fig. 1. The requirement for informed consent was waived due to the retrospective nature of this study. To investigate whether the repeatability and prognostic value of RFs are study-specific or generalizable across different cancer types, we included two distinct cancers, EC and NPC, which differ in their morphological and pathological characteristics. The detailed inclusion and exclusion criteria are presented in Supplemental A1. We finally enrolled 234 EC patients and 525 NPC patients from Xijing Hospital. To evaluate the generalizability of highly repeatable features, an external dataset of 120 EC patients from Sichuan Cancer Hospital was also included. Local recurrence-free survival (LRFS) for patients with EC and distant metastasis-free survival (DMFS) for patients with NPC were collected to evaluate the prognostic performance of RFs. The procedure of treatment and follow-up is described in Supplemental A2. The clinical characteristics and survival outcomes of the included patients are summarized in Table 1. For images, we collected contrast-enhanced computed tomography (CECT) images of all EC and NPC patients, generated by the same scanner. Additionally, fluorine-18 fluorodeoxyglucose positron emission tomography (^18^F-FDG PET) images of EC patients were collected. The imaging protocols are summarized in Supplemental A3.

**Table 1:** The clinical characteristics and survival outcomes of the included patients

Characteristics	EC (Xijing)	EC (Sichuan)	NPC (Xijing)
	*N* = 234	*N* = 120	*N* = 525
Age (year, median [IQR])	68 [62, 73]	62 [56, 66]	48 [41, 55]
Gender (male/female)			
Male	181 (77.4%)	91 (75.8%)	375 (71.4%)
Female	53 (22.6%)	29 (24.2%)	150 (28.6%)
T stage			
1	4 (1.7%)	0 (0%)	57 (10.9%)
2	26 (11.1%)	13 (10.8%)	177 (33.7%)
3	129 (55.1%)	60 (50.0%)	123 (23.4%)
4	75 (32.1%)	47 (39.2%)	168 (32.0%)
N stage			
0	40 (17.1%)	0 (0%)	12 (2.3%)
1	116 (49.6%)	53 (44.2%)	133 (25.3%)
2	61 (26.1%)	59 (49.2%)	273 (52.0%)
3	17 (7.2%)	8 (6.6%)	107 (20.4%)
M stage			
0	234 (100%)	120 (100%)	492 (93.7%)
1	0 (0%)	0 (0%)	33 (6.3%)
Tumor location			
Cervical/upper	57 (24.4%)	58 (48.3%)	–
Middle	80 (34.2%)	46 (38.3%)	
Lower	97 (41.4%)	16 (13.4%)	
Tumor size (median [IQR])	Length (cm): 5.0 [4.0, 7.0]	Length (cm): 5.0 [3.0, 6.1]	Volume (cm^3^): 22.1 [11.1, 41.2]
Treatment	dRT: 18 (7.7%)	dRT: 10 (8.3%)	dCRT: 174 (33.1%)
	dCRT: 216 (92.3%)	dCRT: 110 (91.7%)	IC-dCRT: 351 (66.9%)
Follow-up	LRFS	LRFS	DMFS
Event	128 (54.7%)	79 (65.8%)	111 (21.1%)
Censored	106 (45.3%)	41 (34.2%)	414 (78.9%)
Median survival (months, 95% CI)	27.0 [21,50]	19.7 [12.7, 25.0]	–
3-year survival (%, 95%CI)	43.8 [37.5, 51.2]	31.4 [23.6, 41.8]	83.0 [79.4, 86.7]

Categorical variables were reported as frequencies (proportions). Continuous variables were reported as median (interquartile range [IQR])

*EC* esophageal cancer, *NPC* nasopharyngeal carcinoma, *dRT* definitive radiotherapy, *dCRT* definitive concurrent chemoradiotherapy, *IC* induction chemotherapy, *CECT* contrast-enhanced computed tomography, *PET* positron emission tomography, *LRFS* local recurrence-free survival, *DMFS* distant metastasis-free survival

### Image preprocessing and region segmentation

All images underwent standardized preprocessing. For CECT, a unified mediastinal window (window width 400, window level 40) was used to better reflect tissue anatomical information. For PET, image intensities were normalized to decay-corrected injected activity per kg body weight (SUV [g/mL]). All images were resampled to 1 × 1 × 1 mm^3^ using bspline interpolation. Tumor regions were manually segmented using ITK-SNAP software by a radiologist with 5 years of experience and corrected by two radiologists with 10 years of experience. Peritumoral regions and lymph nodes, which demonstrate prognostic relevance in cancer research [20, 21], were also segmented. All radiologists were strictly blinded to clinical history, pathological diagnoses, and treatment outcomes during the segmentation process. Detailed delineation criteria and methods for peritumoral regions and lymph nodes are provided in Supplemental A4.

### RF extraction

A total of 1316 RFs were extracted from each image using PyRadiomics (version 3.0.1), following the guidelines of the Image Biomarker Standardization Initiative [22]. Specifically, 18 first-order features and 75 textural features were calculated from the original images, 5 Laplacian-of-Gaussian (LoG)-filtered images, and 8 wavelet-filtered images, which were discretized by a fixed bin count of 128 before feature extraction. Fourteen shape-based features were also extracted from the original image. To evaluate the impact of discretization on RF repeatability, additional bin counts (8, 16, 32, and 64) were tested. The parameters of feature extraction are listed in Table S1.

### Assessment of RF repeatability

RF repeatability was assessed via image perturbation, as proposed by Zwanenburg et al [19]. Twenty perturbed images were randomly generated, and RFs were recalculated. RF repeatability was quantified using the one-way, random intraclass correlation coefficient (ICC), with higher values indicating better repeatability. Detailed perturbation parameters and ICC calculation formula are provided in Table S1 and Supplemental A5, respectively.

### Comparison of RF repeatability

Spearman correlation and Mann–Whitney *U*-tests were used to assess consistency and differences in RF repeatability across cancer types, pathological regions, and imaging modalities. The influence of discretization and image preprocessing on repeatability was also evaluated.

### Common independent prognostic RF of EC and NPC

RFs with ICC > 0.75 were identified as highly repeatable, following methodological guidelines for reliability and prior radiomics studies establishing this threshold to ensure robustness against imaging perturbations [18, 23–28]. Common high-repeatable features were selected based on their prognostic performance (Concordance index (*C*-index) > 0.50) in both EC and NPC. Taking into account the correlation between tumor volume and prognosis, the absolute value of the Spearman correlation coefficient quantified the volumetric correlation between each feature and mesh volume, and a threshold of 0.6 to exclude volume-dependent features [6, 29, 30]. Volume-independent features were further assessed using Kaplan–Meier analysis and log-rank tests. Feature redundancy analysis was performed to identify independent prognostic features. The methodological details are provided in Supplemental A6.

### Assessment of RF generalizability

To evaluate whether highly repeatable RFs exhibit superior generalizability across institutions, 234 EC patients from Xijing Hospital (training set) were used to evaluate the performance of RFs for predicting LRFS, and 120 EC patients from Sichuan Cancer Hospital were used as an external testing dataset. Prognostic performance and generalizability were assessed using the *C*-index and Kaplan–Meier analysis. The methodological details are provided in Supplemental A7.

### Assessment of the radiomics quality score (RQS)

The RQS [3] was calculated using an online tool (https://www.radiomics.world/rqs) to evaluate the methodological rigor and reporting standards of this study.

## Results

**Fig. 2:**
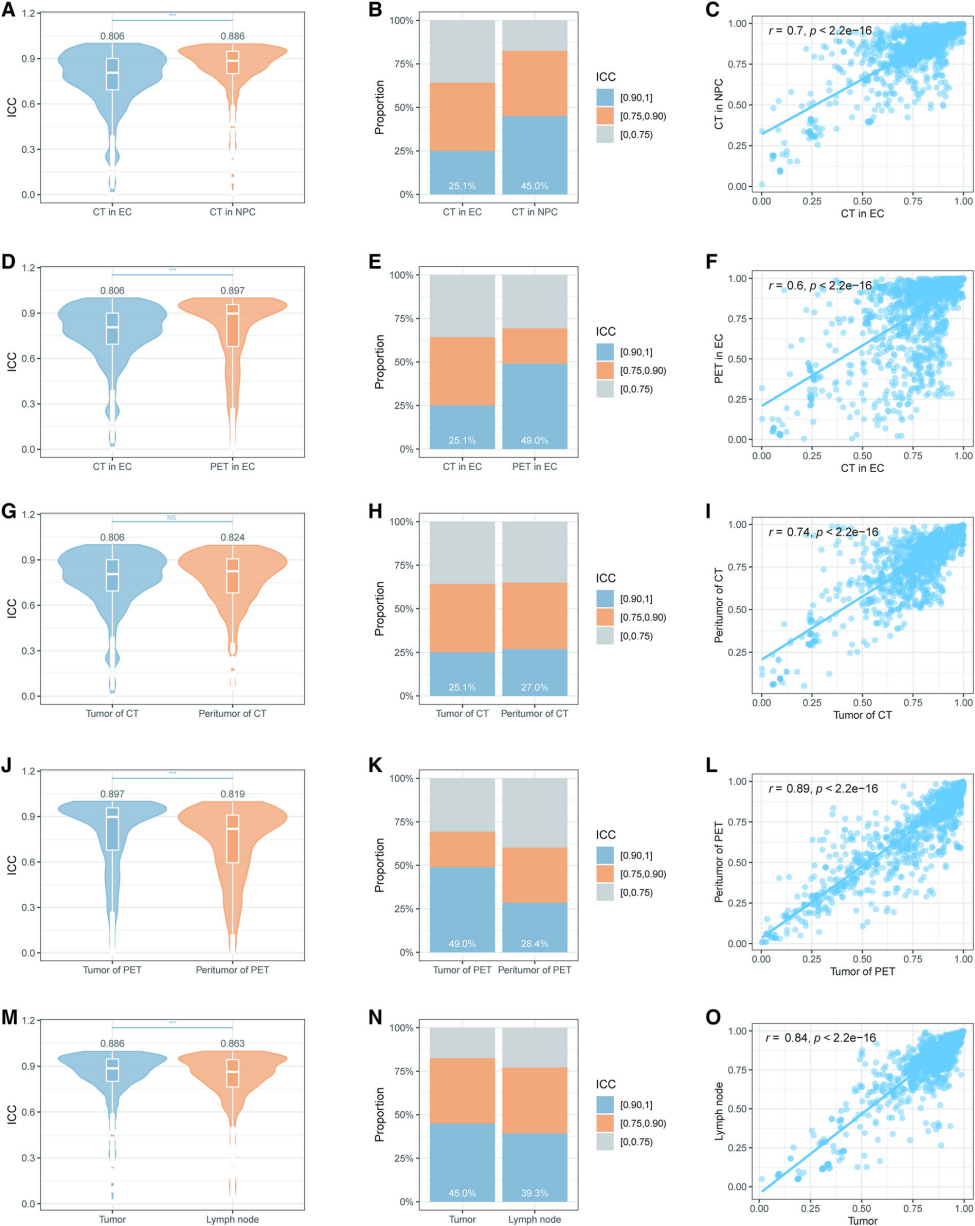
Comparison of RF repeatability across different cancer types, imaging modalities, and pathological regions. **A**–**C** Comparison of RF repeatability between CT-based features in EC and NPC. **D**–**F** Comparison of RF repeatability between CT and PET-based features in EC. **G**–**I** Comparison of RF repeatability between tumor and peritumoral regions in CT-based EC. **J**–**L** Comparison of RF repeatability between tumor and peritumoral regions in PET-based EC. **M**–**O** Comparison of RF repeatability between tumor and lymph node regions in CT-based NPC

The repeatability of RFs demonstrated significant variations across cancer types, pathological regions, and imaging modalities. Notably, CT-based RFs in NPC showed superior repeatability compared to EC (median ICC: 0.886 vs 0.806, *p* < 0.05, Fig. 2A), with nearly double the proportion of highly repeatable features (45.0% vs 25.1%, Fig. 2B). This pattern extended to imaging modalities, where PET-based RFs in EC exhibited significantly higher repeatability than CT-based features (median ICC: 0.897 vs 0.806, *p* < 0.05, Fig. 2D), with 49.0% vs 25.1% highly repeatable features (Fig. 2E). While CT-based peritumoral features in EC demonstrated comparable repeatability (median ICC: 0.824 vs 0.806, *p* > 0.05, Fig. 2G) and proportion of highly repeatable features to tumor features (27.0% vs 25.1%, Fig. 2H), PET peritumoral RFs showed significant degradation in both ICC values (median ICC: 0.819 vs 0.897, *p* < 0.05, Fig. 2J) and proportion of highly repeatable features (28.4% vs 49.0%, Fig. 2K). Similarly, NPC lymph node features exhibited reduced repeatability compared to primary tumors (median ICC: 0.863 vs 0.886, *p* < 0.05, Fig. 2M), with 39.3% vs 45.0% highly repeatable features (Fig. 2N). Although there were differences between the RF repeatability of different cancer types, different pathological regions, and different imaging modalities, feature repeatability was significantly correlated (Bonferroni-corrected *p* < 0.05, Fig. 2C, F, I, L, O).

**Fig. 3:**
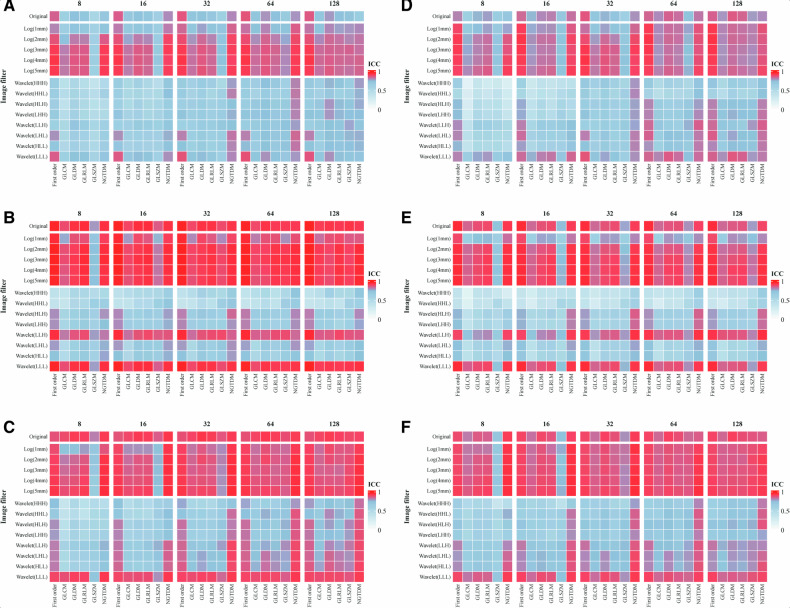
Mean ICC of RFs subgrouped by bin count values, image filters, and feature classes for different cancer types, different imaging modalities, and different pathological regions. The heatmaps in the first column show the mean ICC of subgrouped RFs, which were extracted from the tumor of CT in EC (**A**), PET in EC (**B**), and CT in NPC (**C**). The heatmaps in the second column show the mean ICC of subgrouped RFs, which were extracted from the peritumor of CT (**D**) and PET (**E**) in EC, and the lymph node in NPC (**F**)

To verify the effects of bin count values, filters and feature classes on RF repeatability, the mean ICC values of RFs from different bin counts, different filters, and different feature classes in each imaging dataset are shown in the heatmap of Fig. 3. Overall, the first-order features from the images processed via LoG filtering and a larger bin count were more repeatable, whereas the texture features from the images processed via wavelet filtering and a lower bin count showed lower repeatability. In the workflow of radiomics, a specific bin count is applied to perform intensity discretization for feature extraction. Therefore, we further compared the effects of bin count values of 8, 16, 32, 64, and 128 on feature repeatability (Fig. S1). The RFs from images processed by using a larger bin count exhibited higher repeatability. Moreover, image preprocessing improved the RF repeatability of CT-based tumor in NPC (median ICC: 0.886 vs 0.822, *p* < 0.05), and the details are shown in Fig. S2.

**Fig. 4:**
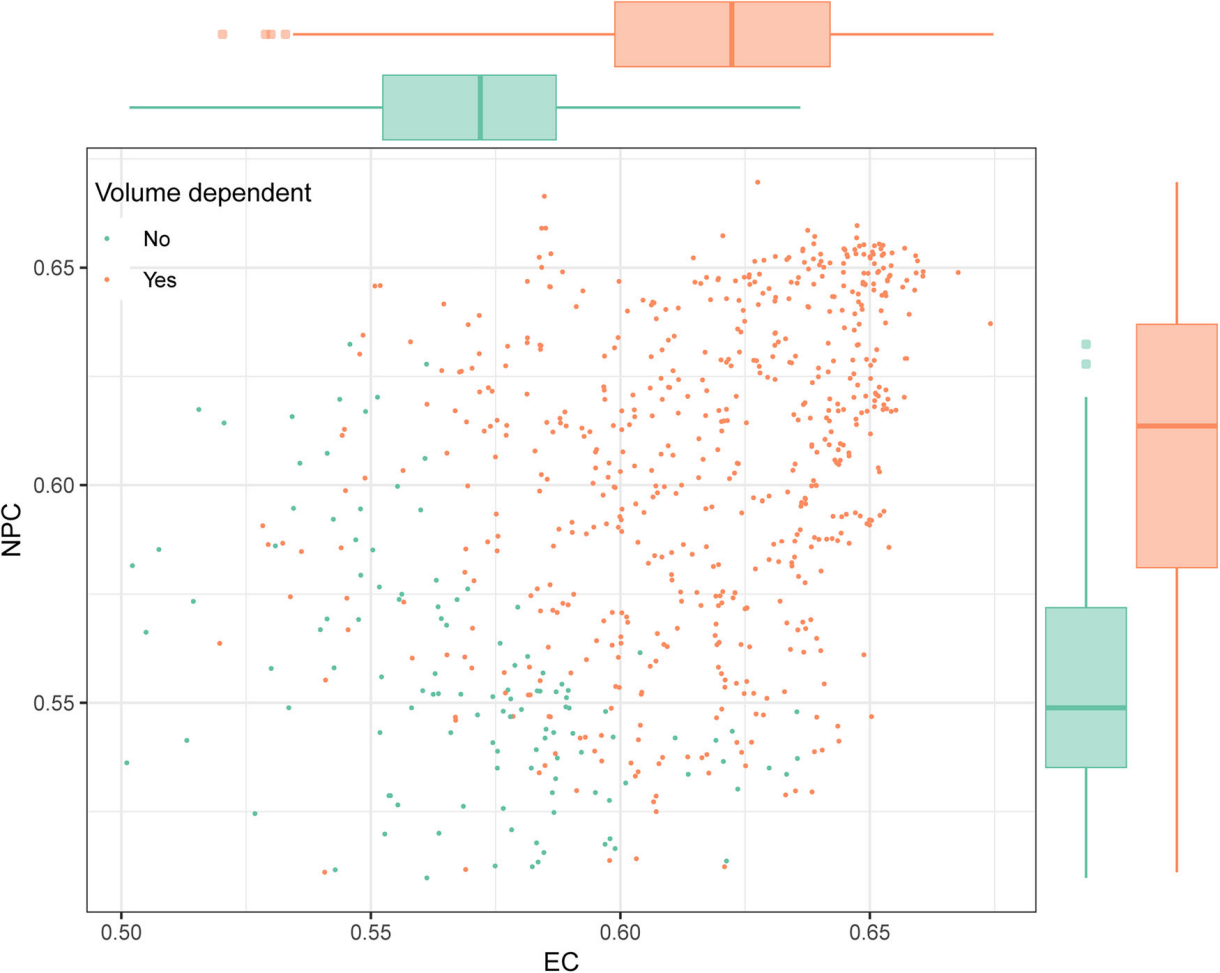
Prognostic performance of the common potential prognostic RFs in EC and NPC. The prognostic performance of volume-dependent RFs was higher than that of the volume-independent RFs

**Fig. 5:**
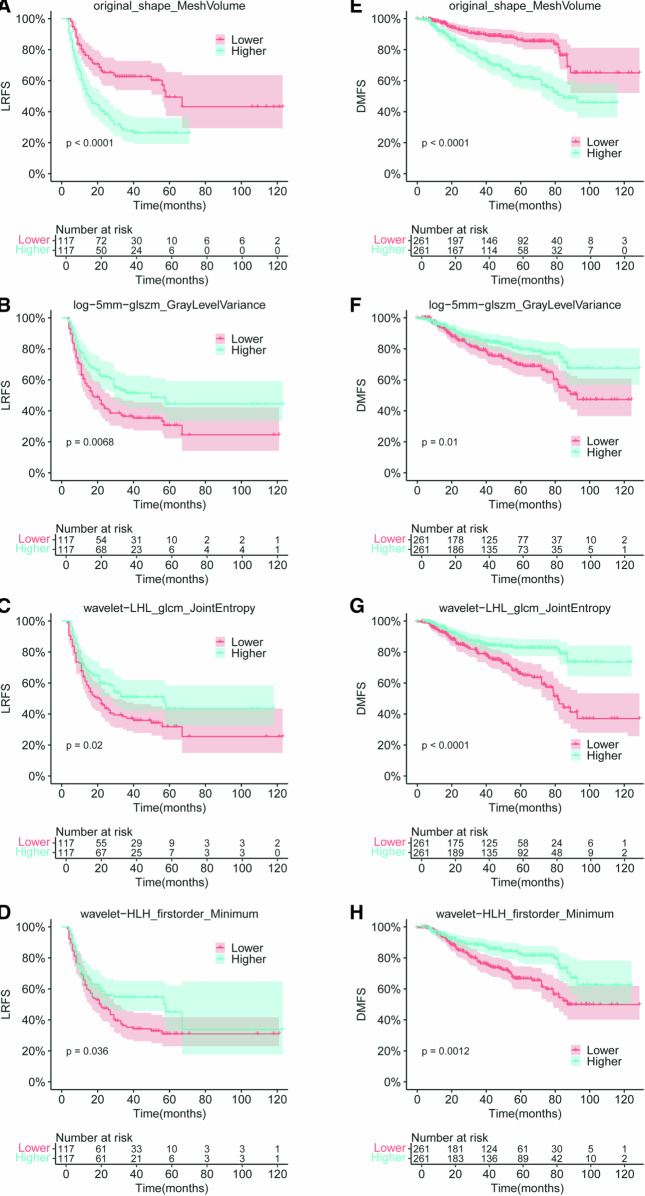
Kaplan–Meier survival analysis of the common independent prognostic features in EC and NPC. The survival curves of the high-risk and low-risk groups divided by the median of each common independent prognostic feature were significantly different in terms of the LRFS of patients with EC (**A**–**D**) and the DMFS of patients with NPC (**E**–**H**)

The prognostic performance of 797 common high-repeatable RFs with ICC > 0.75 in both EC and NPC was evaluated, and the 727 RFs with *C*-index > 0.50 were further selected as the common potential prognostic features, which consisted of 606 volume-dependent RFs and 121 volume-independent RFs (Fig. 4). The prognostic performance of volume-dependent RFs was higher than that of the volume-independent RFs (median *C*-index: 0.62 vs 0.57 in EC, 0.61 vs 0.55 in NPC, Mann–Whitney *U*-test: *p* < 0.05). In addition to the mesh volume, nine common volume-independent prognostic RFs were further selected. The results of the correlation analysis of these features in EC and NPC revealed that some features were highly relevant (|*r*| > 0.6, Fig. S3). Together with the mesh volume, the independent prognostic features are shown in Table 2. The common independent prognostic features of EC and NPC included original_shape_MeshVolume, log-5mm-glszm_GrayLevelVariance, wavelet-LHL_glcm_JointEntropy, and wavelet-HLH_firstorder_Minimum. The survival curves of the high-risk and low-risk groups divided by the median of each common independent prognostic feature are shown in Fig. 5, with significant differences in the LRFS of patients with EC and DMFS of patients with NPC. The *C*-index and hazard ratio (HR) are shown in Table 2.

**Table 2:** List of independent prognostic features in EC and NPC

Cancer type	Filter	Class	Name	ICC	*C*-index	HR [95% CI]
EC	Original	Shape	MeshVolume	0.971	0.648	2.33 [1.64–3.3]
	log-2mm	GLCM	MaximumProbability	0.973	0.578	1.76 [1.24–2.5]
	log-5mm	GLSZM	GrayLevelVariance	0.982	0.564	0.62 [0.44–0.88]
	Wavelet-LHL	GLCM	JointEntropy	0.995	0.551	0.67 [0.47–0.94]
	Wavelet-HLH	Firstorder	Minimum	0.980	0.548	0.69 [0.49–0.98]
NPC	Original	Shape	MeshVolume	0.954	0.655	2.68 [1.85–3.89]
	log-5mm	GLSZM	GrayLevelVariance	0.988	0.572	0.61 [0.42–0.88]
	Wavelet-LHL	GLCM	JointEntropy	0.991	0.585	0.44 [0.3–0.64]
	Wavelet-HLH	Firstorder	Minimum	0.989	0.579	0.53 [0.37–0.78]

*ICC* intraclass correlation coefficient, *C-index* concordance index, *HR* hazard ratio, *CI* confidence interval, *EC* esophageal cancer, *NPC* nasopharyngeal carcinoma, *GLCM* gray level co-occurrence texture matrix, *GLSZM* gray level size zone matrix

In total, 234 patients with EC at Xijing Hospital and 120 patients with EC at Sichuan Cancer Hospital were used to assess whether highly repeatable features have better generalizability. The prognostic performance of RFs from the high-repeatable RF group and low-repeatable RF group in the training and testing sets is shown in Fig. S4A, B. Compared with the low-repeatable RF group, the high-repeatable RF group had better prognostic performance (median *C*-index: 0.614 vs 0.578 in training and 0.603 vs 0.562 in testing, *p* < 0.05). Notably, the high-repeatable group had more stable generalizability (median|△*C*-index|: 0.022 vs 0.029, *p* < 0.05). In addition, the external testing set was also divided into high- and low-risk groups by the median of the independent prognostic features in the training set (Fig. S5). The LRFS of the two groups divided by original_shape_MeshVolume and wavelet-HLH_firstorder_Minimum was significantly different (HR [95% confidence interval (CI)]: 1.97 [1.26–3.06] and 0.61 [0.39–0.95]).

The detailed calculation process and corresponding RQS are presented in Table S2. A 100% score is reached at 36 points, and the RQS for this study was 11.

## Discussion

This study comprehensively evaluated the repeatability and prognostic value of RFs across different cancer types (EC and NPC), pathological regions (tumor, peritumor, and lymph node), and imaging modalities (CT and PET). The key findings revealed that RF repeatability was influenced by cancer type, pathological region, and imaging modality, while certain features maintain consistent prognostic performance across different cancer types. These findings provide valuable insights into the generalizability of radiomics models and their potential application in multi-cancer prognostic modeling.

Previous studies on RF repeatability have primarily focused on a single cancer type, limiting the generalizability of their findings. For instance, studies by Zhang et al [5] and Fiset et al [9]. investigated RF repeatability in NPC and cervical cancer, respectively, but did not compare across different cancer types. Janna et al investigated the stability of radiomics features via test–retest analyses on CT scans of rectal and lung cancer patients, highlighting the need for disease-specific assessment to identify robust features [11]. Owing to the simultaneous differences in hardware, scan acquisition and reconstruction settings, disease sites, and scan time intervals in their study, independently assessing the impact of each factor on feature repeatability is difficult. In this study, we controlled for scanner variables to reveal the differences in RF repeatability between EC and NPC.

Our findings demonstrated that peritumoral RFs in CT exhibited superior reproducibility compared to intratumoral features, aligning with prior studies by Tunali et al [31], who attributed this phenomenon to the relatively homogeneous microenvironment in peritumoral regions vs the intrinsic heterogeneity of tumor cores. However, the reversed trend observed in PET imaging warrants further exploration. This discrepancy may stem from fundamental differences in imaging physics and biological correlates: CT predominantly reflects anatomical density variations, whereas PET quantifies metabolic activity. In tumors, metabolic heterogeneity within the core (e.g., hypermetabolic foci) may paradoxically enhance feature stability in PET due to standardized uptake value (SUV) normalization protocols, whereas peritumoral regions in PET often encompass metabolically ambiguous zones (e.g., necrosis, inflammatory activity, or microscopic invasion), amplifying measurement variability. These modality-specific nuances highlight the importance of context-aware radiomic modeling and underscore the need for standardized preprocessing pipelines tailored to imaging modalities.

In this study, we evaluated the consistency of the effects of different bin counts, different filters, and different feature classes on RF repeatability in different cancer types, different pathological regions, and different imaging modalities. Reducing the bin count may amplify differences in texture features as a consequence of the diminished size of the gray-level matrix [5]. Wichtmann et al indicated that at least 32 bins should be employed for MRI, and the intensity discretization to 64 bins might rarely lead to more repeatable features [32]. Moreover, too large a bin count may introduce too much noise. We also explored the effect of bin count on RF repeatability in different regions of PET and CT, suggesting a reasonable selection for specific studies. The repeatability of features derived from wavelet filtering with high-pass filters was consistently poor across datasets, as these features predominantly retain high-frequency components of the image, such as edges and textural details, which are highly sensitive to minute variations in the image, thereby diminishing the repeatability of associated features [11, 31]. Conversely, the LoG filtering, by virtue of its smoothing-before-enhancement characteristic, effectively mitigates noise interference, leading to more stable feature extraction. In particular, with the increase of scale parameter (sigma), the image experiences greater smoothing, which enhances robustness against noise and minor alterations [33]. Compared with first-order features, which focus on the global gray distribution of the image, texture features analyze pixel relations and are sensitive to local changes and noise, resulting in poorer feature repeatability. These consistent findings provide broader validation for previous studies.

This study provides evidence that high repeatability guarantees the external generalizability of prognostic features, and further reveals that high-repeatable RFs might have prognostic performance across cancer types. In machine learning, overfitting refers to the phenomenon where a model performs well on training data but degrades on new data, often due to the model overlearning noise or chance in the training data. Features with high repeatability could provide consistent information across patients facing the same clinical situation or across different healthcare facilities, which means that these features are less likely to be affected by random image noise. Therefore, using high-repeatable features can reduce the possibility of such overreliance on specific noise or details in the training data, thereby reducing the risk of overfitting. Because of their consistency under different conditions, the high-repeatable features not only help the model learn more general rules but also reduce the misjudgment caused by accidental factors, which is the key to realizing the generalizability of the model to new data and new environments. Our results are consistent with those of previous studies showing that high-repeatable RFs enhance model generalizability [4–6].

Notably, we identified several high-repeatable features with consistent prognostic ability in both EC and NPC. These features may touch on core mechanisms of tumor biology that are prevalent across multiple tumor types and thus have broad predictive value. Volume is an important independent prognostic factor for both EC and NPC, and reflects the overall condition of the tumor. Chang et al proposed that tumor volume had a greater effect on the prognosis of NPC than T stage [34]. Kang et al also suggested that tumor volume based on CT imaging was superior to the T stage of tumor invasion depth in predicting the prognosis of nonoperative EC patients [35]. The larger the tumor size, the greater the burden of tumor cells, the more intratumoral blood flow disorders, and the more hypoxic cells. These factors might affect the theatment efficacy, leading to local uncontrolled disease or recurrence after treatment, thus affecting long-term survival. Further assessment of the biological significance of these prognostic features in different cancer types via radiogenomics is encouraged. Our results also demonstrated that the prognostic performance of volume-dependent RFs was significantly higher than that of volume-independent RFs. This observation aligns with the findings of Traverso et al [36], who reported that volume-related RFs played a predominant role in prognostic models for lung and head-and-neck cancers, whereas volume-independent RFs lacked sufficient independent prognostic power. Certain shape-based features (e.g., SurfaceArea) essentially quantify tumor dimensional characteristics, serving as sophisticated measurements of tumor bulk. While clinically valuable, these features may not provide substantial new biological information beyond conventional size assessments. Notably, some volume-associated texture features (e.g., LargeAreaEmphasis) may encode additional biological information by capturing spatial heterogeneity patterns across tumor volumes or regional microenvironmental variations. Such features might represent a convergence of volumetric and textural information, where tumor size serves as a scaffold for spatial heterogeneity patterns. Moreover, volume-independent features retain value by detecting localized biological processes, and their integration with volume-dependent features provides more comprehensive prognostic information through complementary tumor characterization.

This study has several limitations. First, although perturbation analysis is a feasible alternative to test–retest imaging, perturbations may not fully capture all the real-world variability encountered in clinical practice. Second, while the experimental design evaluated the repeatability of RFs under identical imaging conditions, their reproducibility across heterogeneous clinical settings remains to be validated. Third, our dataset lacked diversity in patient demographics (e.g., age, gender, and ethnic distributions), which may limit the generalizability of our findings across different populations. Finally, although the RQS score of 11 aligns with the average quality of existing radiomics studies in EC (9.07) [37], there are several aspects that need to be improved in future work: (1) multivariable integration: combining RFs with established prognostic factors (for example, TNM-staging) to develop holistic prediction systems; (2) biological interpretation: exploring associations between stable RFs and underlying gene-protein expression patterns through radiogenomics to deepen understanding of radiomics and biology; and (3) clinical validation: conducting prospective trials to provide the highest level of evidence supporting the clinical validity and usefulness of the radiomics biomarker. These improvements would provide high-quality radiomic biomarkers and transform radiomic models from research tools to clinically actionable decision-support systems.

## Conclusions

In this study, we found that RF repeatability exhibits significant heterogeneity across cancer types, imaging modalities, and pathological regions, while certain features demonstrate robust prognostic generalizability regardless of tumor origin. These findings underscore that in the practice of radiomics for specific clinical or research objectives, systematic assessment of RF repeatability is not only a necessary step, but also essential to ensure the generalizability of prognostic signatures across centers and diseases. This study advances the generality and practicality of radiomics in diverse clinical scenarios, facilitating the progression of precision medicine.

## Supplementary information

ELECTRONIC SUPPLEMENTARY MATERIAL

## Data Availability

Data are available from the corresponding author upon request.

## Abbreviations

*C*-index: Concordance index
CECT: Contrast-enhanced computed tomography
DMFS: Distant metastasis-free survival
EC: Esophageal cancer
GLCM: Gray level co-occurrence texture matrix
GLSZM: Gray level size zone matrix
ICC: Intraclass correlation coefficient
LoG: Laplacian-of-Gaussian
LRFS: Local recurrence-free survival
NPC: Nasopharyngeal carcinoma
RF: Radiomic features
RQS: Radiomics quality score
