# Supplementary material for: Repeatability and prognostic value of radiomic features: a study in esophageal cancer and nasopharyngeal carcinoma
Source: Insights Imaging. 2025 Aug 2;16:166. doi: 10.1186/s13244-025-02044-z (PMC12316612; doi:10.1186/s13244-025-02044-z)

Repeatability and Prognostic Value of Radiomic Features: A Study in Esophageal Cancer and Nasopharyngeal Carcinoma

ELECTRONIC SUPPLEMENTARY MATERIAL

Supplemental A1. Detailed inclusion and exclusion criteria.

This study enrolled patients with locally advanced esophageal squamous cell cancer (ESCC) who received definitive (chemo)radiotherapy. The inclusion and exclusion criteria are as follows: Inclusion criteria: (1) pathologically confirmed ESCC; (2) contrast-enhanced CT (PET) within 1 week before treatment; (3) definitive radiation dose ≥50 Gy; (4) the follow-up time for all patients after treatment exceeded two years. Exclusion criteria: (1) distant metastatic disease; (2) patients received esophagectomy or preoperative/postoperative adjuvant radiotherapy; (3) lack of clinicopathological data or follow-up information; (4) died within three months after treatment; (5) low-quality CT or PET imaging.

This study enrolled patients with nasopharyngeal carcinoma (NPC) who received definitive concurrent chemoradiotherapy (with or without induction chemotherapy). The inclusion and exclusion criteria are outlined below: Inclusion Criteria: (1) pathologically confirmed NPC; (2) contrast-enhanced CT within 1 week before treatment; (3) definitive radiation dose ≥70 Gy; (4) the follow-up time for all patients after treatment exceeded two years. Exclusion Criteria: (1) history of prior nasopharyngeal surgery; (2) concomitant history of other malignancies; (3) lack of clinicopathological data or follow-up information; (4) died within three months after treatment; (5) low-quality CT imaging.

Supplemental A2. The procedure of treatment and follow-up.

Definitive chemoradiotherapy has become the current standard treatment for unresectable EC[1]. The definition and dose prescription of radiotherapy target volumes and dose constraints to the organs at risk followed the protocols reported by our previous study[2]. A total prescribed dose of 50-70 Gy to the planning target volume (PTV) in 25 to 35 fractions was delivered to the patients. For chemotherapy regimen, more than half of the patients received paclitaxel and cisplatin (TP) regimen, Fluorouracil/capecitabine and cisplatin (PF) regimen, or cisplatin and S1 (CS) regimen. Part of patients received capecitabine or S1. The choice of chemotherapy was based on multidisciplinary

team decision and preference of patients. After treatment, follow-up evaluations were performed once a month in the first year, every 3 months for the first 2 years, every 6 months for 3-5 years, and once a year thereafter. Each evaluation included the physical examination, the blood test, the barium esophagram, the CT scan of the neck and chest, and the abdominal ultrasound. The endoscopy and biopsy were performed once local recurrence was suspected. Patients were followed up for more than 2 years after treatment. LRFS was defined as the time from the first date of therapy until the earliest occurrence of local recurrence or death.

Moreover, definitive concurrent chemoradiotherapy (with or without induction chemotherapy) is a standard of care for locally advanced NPC[3-5]. A definitive radiation dose of 70–72 Gy in 33 fractions was prescribed to the primary tumor (PGTVnx), while metastatic lymph nodes (PGTVnd) received 68–70 Gy in 33 fractions. High risk and low risk clinical target volumes received 60 Gy and 54-56 Gy in 28-33 fractions. Concurrent chemotherapy consisted of cisplatin. For cisplatin-intolerant patients, nedaplatin was substituted. Induction chemotherapy primarily employed gemcitabine + cisplatin (GP regimen). Post-treatment follow-up was conducted every 3 months in the first 2 years, every 6 months during 3–5 years, and annually thereafter. Each evaluation included routine blood tests (complete blood count, liver/kidney function, EBV-DNA quantification, thyroid function), fiberoptic examination with narrow band imaging, neck lymph node ultrasound, and chest CT. Nasopharyngeal and neck

MRI was performed biannually or as needed, and ^{99m}Tc-MDP scans were conducted annually. If recurrence or metastasis was suspected, biopsy or PET-CT scan was performed for confirmation. Patients were followed up for more than 2 years after treatment. DMFS was defined as the time from the first date of therapy until the earliest occurrence of distant metastasis or death.

Supplemental A3. The imaging protocols for each center, modality, and tumor types.

The contrast-enhanced CT imaging protocol for EC at Xijing Hospital utilized a Philips Brilliance Big Bore scanner with the following parameters: tube voltage, 120 kVp; tube current, 200-250 mAs; acquisition matrix, 512×512 mm; field of view, 350×350 mm; detector collimation, 16×1.5 mm; rotation time, 0.75 s; slice thickness, 5 mm; Standard B (body) reconstruction kernel. For contrast enhancement, iodinated contrast agent was administered intravenously at 1.5 mL/kg body weight with an injection rate of 3.0 mL/s. Venous phase images were acquired after a 70-second delay.

The PET imaging protocol for EC at Xijing Hospital utilized a Siemens Biograph40 scanner with scan coverage from vertex to upper third of femur. ^{18}F-FDG with radiochemical purity >95% was used as the tracer. Patients fasted for ≥6 hours and maintained blood glucose levels <11 mmol/L prior to intravenous administration of 5.60-7.40 MBq/kg ^{18}F-FDG in a quiet, dimly lit environment. Whole-body PET/CT acquisition commenced 60 minutes post-

injection, covering 5-6 bed positions at 70 s per bed position. The scan settings included a tube voltage of 120 kV, a tube current of 100 mA, and a slice thickness of 3 mm. PET images were reconstructed via ordered subsets expectation maximization algorithm and corrected for attenuation using the CT dataset.

The contrast-enhanced CT imaging protocol for NPC at Xijing Hospital utilized a Philips Brilliance Big Bore scanner with the following parameters: tube voltage, 120 kVp; tube current, 350-395 mAs; acquisition matrix, 512×512 mm; field of view, 350×350 mm; detector collimation, 16×1.5 mm; rotation time, 1.0 s; slice thickness, 3 mm; Standard B (head) reconstruction kernel. For contrast enhancement, iodinated contrast agent was administered intravenously at 1-1.5 mL/kg body weight with an injection rate of 2.0-2.5 mL/s. Venous phase images were acquired after a 40-second delay.

The contrast-enhanced CT imaging protocol for EC at Sichuan Cancer Hospital utilized a Philips Brilliance Big Bore scanner with the following parameters: tube voltage, 120 kVp; tube current, 200-250 mAs; acquisition matrix, 512×512 mm; field of view, 350×350 mm; detector collimation, 16×1.5 mm; rotation time, 0.75 s; slice thickness, 5 mm; Standard B (body) reconstruction kernel. For contrast enhancement, iodinated contrast agent was administered intravenously at 1.5 mL/kg body weight with an injection rate of 2.5 mL/s. Venous phase images were acquired after a 75-second delay.

Supplemental A4. Detailed delineation criteria and methods for peritumoral regions and lymph nodes.

The peritumoral region was defined as a 5 mm expansion around the tumor boundary. To ensure consistency in delineation, a Python script utilizing the "ndimage.binary_dilation" function from the "scipy" library was employed to generate an initial peritumoral region. This initial region was then manually refined by experienced radiologists using ITK-SNAP software to exclude air cavities, adjacent organs, and large vessels. This combined approach of automated expansion and manual refinement ensured that the peritumoral region was delineated as accurately and consistently as possible. Furthermore, the delineation criteria of positive lymph nodes is as follows: ①Retropharyngeal lymph nodes with a minimum diameter of >4mm on transverse imaging; ②Lymph nodes with a minimum diameter of ≥10 mm and level II nodes with a minimum diameter of ≥11 mm on transverse imaging; ③Clustered aggregation of three or more lymph nodes within the same region, with a minimum diameter of ≥5 mm; ④Lymph nodes with central necrosis or contrast enhancement as well as extracapsular invasion.

Supplemental A5. ICC calculation.

The repeatability of each feature was quantified via the one-way, random intraclass correlation coefficient (ICC) [6-8], calculated as follows:

$$\mathrm{ICC} = \frac{MS_R - MS_W}{MS_R + (k-1) * MS_W}$$

where MS_R represents the mean square of average perturbation values for patients, MS_W is the residual source of variance, which is calculated as the variance of perturbation values averaged across patients, and k is the number of perturbations. The ICC ranges from 0 to 1, with higher values indicating better repeatability.

Supplemental A6. The methodological details for identifying common high-repeatable RF of EC and NPC.

For EC and NPC tumors, we screened RFs using ICC>0.75 and identified common high-repeatable features at the intersection. The average of concordance index (C-index) was calculated based on five times of 10-fold cross-validation to evaluate the prognostic performance of each common high-repeatable RF in EC and NPC. The features with C-index greater than 0.50 in both EC and NPC were further selected as the common potential prognostic features. Because tumor volume is often associated with prognosis, we used the absolute value of the spearman correlation coefficient to quantify the volumetric correlation of each feature with mesh volume and a threshold of 0.6 to exclude volume-dependent features. For features that were volume-independent in both EC and NPC, we further assessed the risk stratification

ability using the Kaplan-Meier analysis and the log-rank test to obtain common prognostic features. The feature redundancy analysis was further performed to screen the independent prognostic features from the set of prognostic features in EC and NPC, respectively. Specifically, the spearman correlation was used to assess the correlation of every two RFs. For feature pairs with correlation coefficients greater than 0.6, the feature with a stronger average correlation with other features was removed. The common independent prognostic features were obtained by intersecting the independent prognostic features of EC and NPC.

Supplemental A7. The methodological details of RF generalizability assessment.

To evaluate whether highly repeatable RFs exhibit superior generalizability across institutions, 234 EC patients from Xijing Hospital (training set) were used to evaluate performance of RFs for predicting LRFS, and 120 EC patients from Sichuan Cancer Hospital were used as an external testing dataset. Z-score normalization was applied to all RFs, where the mean and standard deviation were computed exclusively on the training set. These derived parameters were then used to standardize both the training set and the testing set. The C-index was used to assess the prognostic performance of each RF. RF generalizability was evaluated using the absolute value of the difference between the C-index of training and that of testing (|△C-index|). The median of all ICC values in the primary set was used as the threshold to divide all features into a high-

repeatable RF group and a low-repeatable RF group. The Mann-Whitney U test was used to compare the differences in prognostic performance and generalizability between the two groups.

To assess the generalizability of the common independent prognostic features mentioned above, the median of each independent prognostic feature of the training set was used as the threshold to further divide patients in the external testing set into a high-risk group and a low-risk group. The Kaplan-Meier analysis was applied and the *p* value of the log-rank test was used to evaluate the performance of the risk stratification.

References

1 Cooper JS, Guo MD, Herskovic A et al (1999) Chemoradiotherapy of locally advanced esophageal cancer: long-term follow-up of a prospective randomized trial (RTOG 85-01). Radiation Therapy Oncology Group. Jama 281:1623-1627

2 Zhao L, Zhou Y, Pan H et al (2017) Radiotherapy Alone or Concurrent Chemoradiation for Esophageal Squamous Cell Carcinoma in Elderly Patients. J Cancer 8:3242-3250

3 Al-Sarraf M, LeBlanc M, Giri PG et al (1998) Chemoradiotherapy versus radiotherapy in patients with advanced nasopharyngeal cancer: phase III randomized Intergroup study 0099. J Clin Oncol 16:1310-1317

4 Blanchard P, Lee A, Marguet S et al (2015) Chemotherapy and radiotherapy in nasopharyngeal carcinoma: an update of the MAC-NPC meta-analysis. Lancet Oncol 16:645-655

5 Cao SM, Yang Q, Guo L et al (2017) Neoadjuvant chemotherapy followed by concurrent chemoradiotherapy versus concurrent chemoradiotherapy alone in locoregionally advanced nasopharyngeal carcinoma: A phase III multicentre randomised controlled trial. Eur J Cancer 75:14-23

6 Teng X, Zhang J, Ma Z et al (2022) Improving radiomic model reliability using robust features from perturbations for head-and-neck carcinoma. Front Oncol 12:974467

7 Shrout PE, Fleiss JL (1979) Intraclass correlations: uses in assessing rater reliability. Psychol Bull 86:420-428

8 Xue C, Yuan J, Lo GG et al (2021) Radiomics feature reliability assessed by intraclass correlation coefficient: a systematic review. Quant Imaging Med Surg 11:4431-4460

Table S1. The parameters of RF extraction and image perturbation.

Processing	Parameters	Adopted value
RF extraction	Image discretization bin count	8, 16, 32, 64, 128
	Image filters	Original, LoG, Wavelet
	Kernel size of LoG filter (mm)	[1,2,3,4,5]
	Wavelet filter decompositions	[LLL, HLL, LHL, LLH, LHH, HLH, HHL, HHH]
	Feature class	First-order (18), GLCM (24), GLDM (14), GLRLM (16), GLSZM (16), NGTDM (5)
Perturbation	Rotation angles (degree)	[-10,-5,0,5,10]
	Translation distance (mm)	[0,0.2,0.4,0.6,0.8,1]
	Contour randomization (mm)	10

Abbreviations: RF, radiomic feature; Laplacian-of-Gaussian, LoG; L, low-pass; H, high-pass. gray level co-occurrence texture matrix, GLCM; gray level dependence matrix, GLDM; gray level run-length texture matrix, GLRLM; gray level size zone matrix, GLSZM; neighborhood gray tone difference matrix, NGTDM.

Table S2. The RQS of this study.

Image protocol quality - well-documented image protocols (for example, contrast, slice thickness, energy, etc.) and/or usage of public image protocols allow reproducibility/replicability<br>☑ protocols well documented<br>☐ public protocol used<br>☐ none
Multiple segmentations - possible actions are: segmentation by different physicians/algorithms/software, perturbing segmentations by (random) noise, segmentation at different breathing cycles. Analyse feature robustness to segmentation variabilities<br>◉ yes ○ no
Phantom study on all scanners - detect inter-scanner differences and vendor-dependent features.<br>Analyse feature robustness to these sources of variability<br>○ yes ◉ no
Imaging at multiple time points - collect images of individuals at additional time points. Analyse feature robustness to temporal variabilities (for example, organ movement, organ expansion/shrinkage)<br>○ yes ◉ no
Feature reduction or adjustment for multiple testing - decreases the risk of overfitting. Overfitting is inevitable if the number of features exceeds the number of samples. Consider feature robustness when selecting features<br>◉ Either measure is implemented<br>○ Neither measure is implemented
Multivariable analysis with non radiomics features (for example, EGFR mutation) - is expected to provide a more holistic model. Permits correlating/inferencing between radiomics and non radiomics features<br>○ yes ◉ no
Detect and discuss biological correlates - demonstration of phenotypic differences (possibly associated with underlying gene–protein expression patterns) deepens understanding of radiomics and biology<br>○ yes ◉ no
Cut-off analyses - determine risk groups by either the median, a previously published cut-off or report a continuous risk variable. Reduces the risk of reporting overly optimistic results<br>◉ yes ○ no

Discrimination statistics - report discrimination statistics (for example, C-statistic, ROC curve, AUC) and their statistical significance (for example, p-values, confidence intervals). One can also apply resampling method (for example, bootstrapping, cross-validation)<br>☑ a discrimination statistic and its statistical significance are reported<br>☑ a resampling method technique is also applied<br>☐ none
Calibration statistics - report calibration statistics (for example, Calibration-in-the-large/slope, calibration plots) and their statistical significance (for example, P-values, confidence intervals). One can also apply resampling method (for example, bootstrapping, cross-validation)<br>☐ a calibration statistic and its statistical significance are reported<br>☐ a resampling method technique is applied<br>☑ none
Prospective study registered in a trial database - provides the highest level of evidence supporting the clinical validity and usefulness of the radiomics biomarker<br>○ yes ◉ no
Validation - the validation is performed without retraining and without adaptation of the cut-off value, provides crucial information with regard to credible clinical performance<br>☐ No validation<br>☐ validation is based on a dataset from the same institute<br>☑ validation is based on a dataset from another institute<br>☐ validation is based on two datasets from two distinct institutes<br>☐ the study validates a previously published signature<br>☐ validation is based on three or more datasets from distinct institutes
Comparison to 'gold standard' - assess the extent to which the model agrees with/is superior to the current 'gold standard' method (for example, TNM-staging for survival prediction). This comparison shows the added value of radiomics<br>○ yes ◉ no
Potential clinical utility - report on the current and potential application of the model in a clinical setting (for example, decision curve analysis).<br>○ yes ◉ no
Cost-effectiveness analysis - report on the cost-effectiveness of the clinical application (for example, QALYs generated)<br>○ yes ◉ no

Open science and data - make code and data publicly available. Open science facilitates knowledge transfer and reproducibility of the study<br>☐ scans are open source<br>☐ region of interest segmentations are open source<br>☐ the code is open sourced<br>☐ radiomics features are calculated on a set of representative ROIs and the calculated features and representative ROIs are open source
**Total score** **11** (30.56%)

Figure S1. Effect of bin count on the repeatability of RFs extracted from different cancer types, different imaging modalities, and different pathological regions. Based on the ICC of all features extracted from tumors of CT in EC with bin count values of 8, 16, 32, 64 and 128 (A), the violin plots depict the distribution and statistical comparison of RF repeatability based on different bin count values (B), and the percentage bar plots illustrate the proportions of high-repeatable features in groups with different bin count values (C). The effect was also assessed in tumors of PET in EC (D-F), CT in NPC (G-I), and peritumors of CT in EC (J-L), peritumors of PET in EC (M-O), lymph nodes of CT in NPC (P-R).

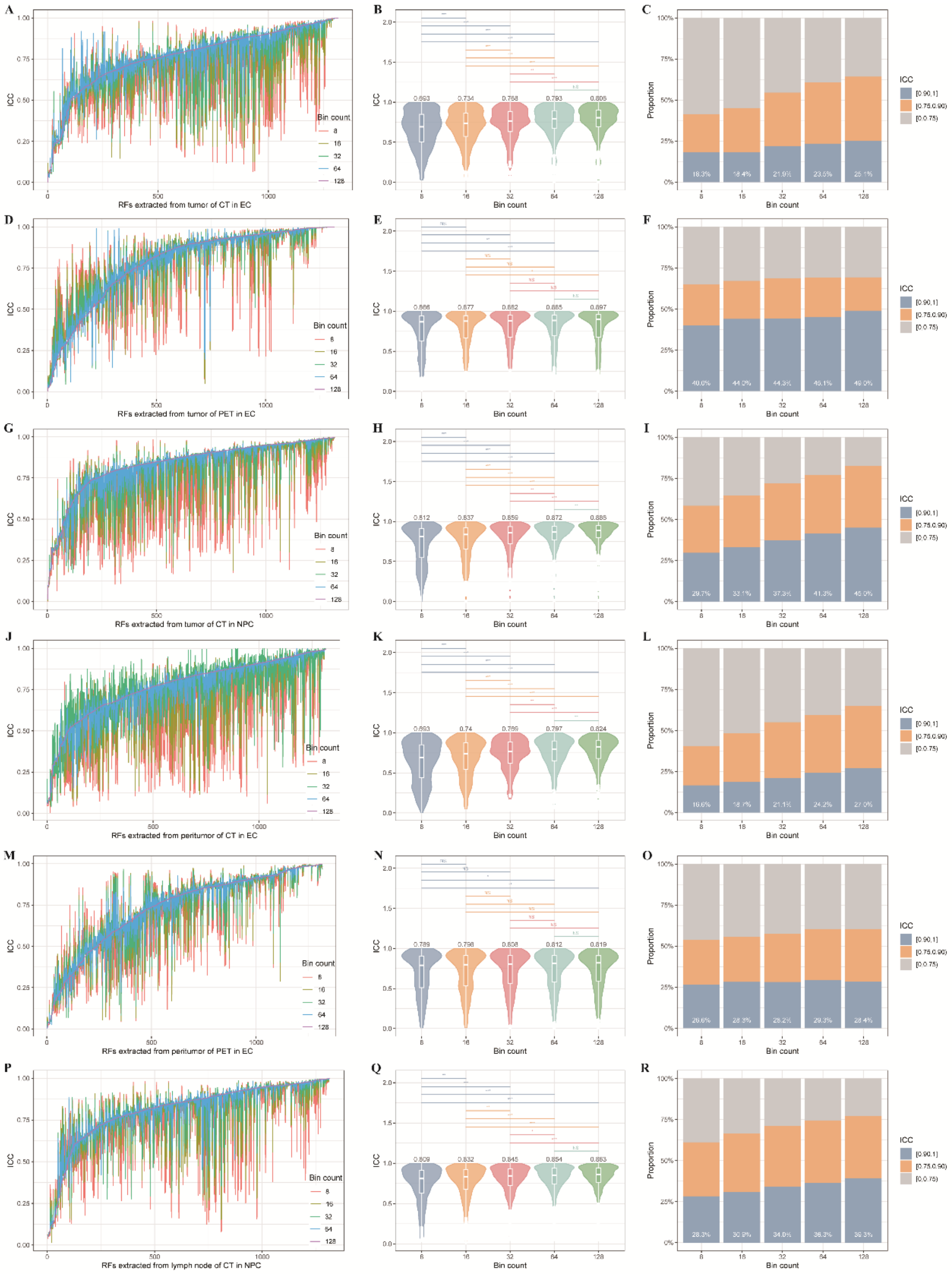
A
B
C
RFs extracted from tumor of CT in EC
D
E
F
RFs extracted from tumor of PET in EC
G
H
I
RFs extracted from tumor of CT in NPC
J
K
L
RFs extracted from peritumor of CT in EC
M
N
O
RFs extracted from peritumor of PET in EC
P
Q
R
RFs extracted from lymph node of CT in NPC
ICC
Bin count
Proportion

Figure S2. Effect of image preprocessing on RF repeatability from tumor of CT in NPC. The heatmap shows the mean ICC of RFs from raw images, which were subgrouped by bin count values, image filters, and feature classes (A). The violin plot depicts the distribution and statistical comparison of RF repeatability for tumors of raw images and processed images based on the bin count of 128 (B). The percentage bar plot illustrates the proportions of high-repeatable features in the two groups (C). The line plot shows the ICCs of all RFs extracted from the original plot and the processed plot (D).

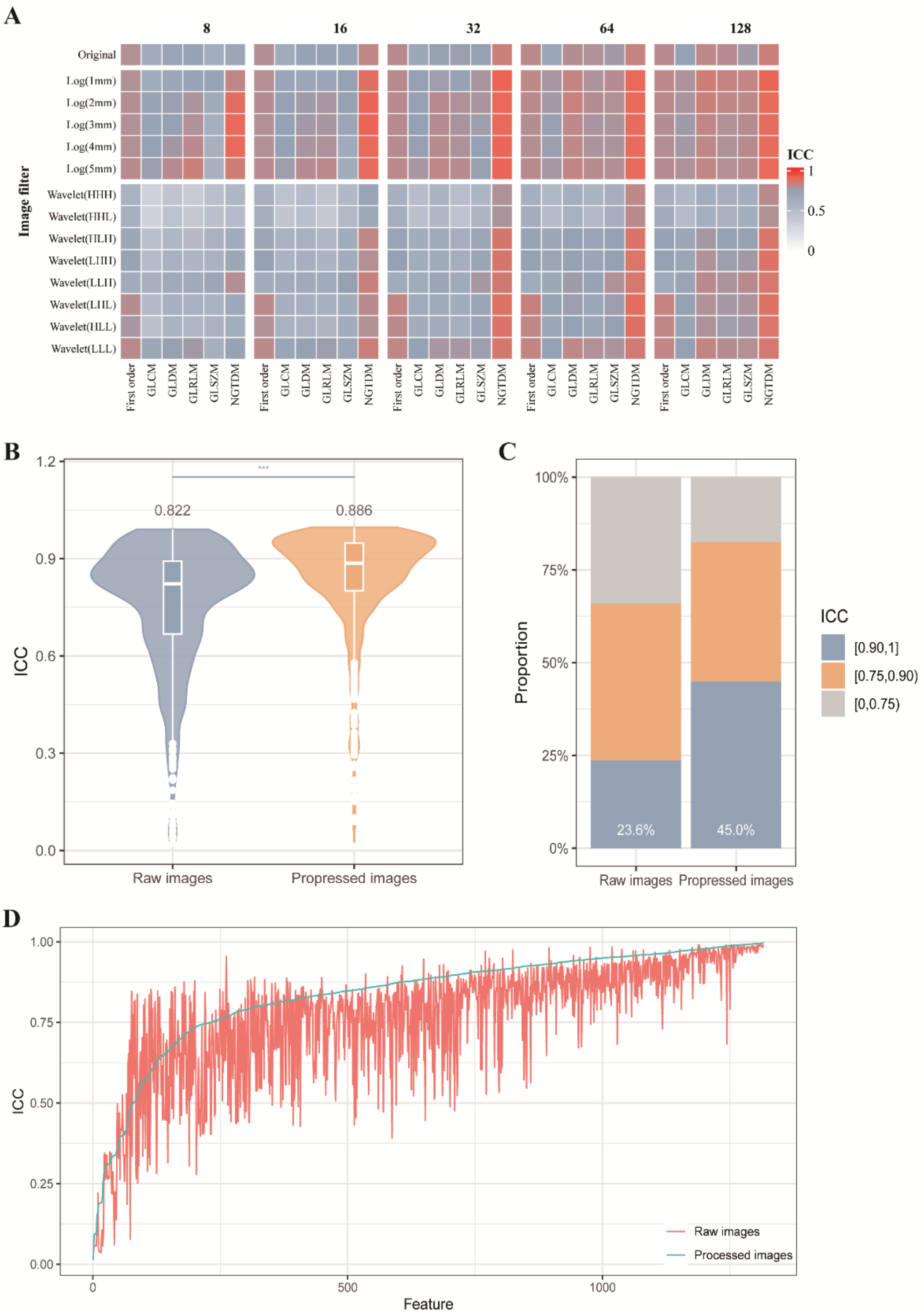
A
8
16
32
64
128
Image filter
Original
Log(1mm)
Log(2mm)
Log(3mm)
Log(4mm)
Log(5mm)
Wavelet(HHH)
Wavelet(HHL)
Wavelet(HLH)
Wavelet(LHH)
Wavelet(LLH)
Wavelet(LHL)
Wavelet(HLL)
Wavelet(LLL)
First order
GLCM
GLDM
GLRLM
GLSZM
NGTDM
ICC
1
0.5
0
B
***
0.822
0.886
ICC
1.2
0.9
0.6
0.3
0.0
Raw images
Propressed images
C
Proportion
100%
75%
50%
25%
0%
23.6%
45.0%
Raw images
Propressed images
ICC
[0.90,1]
[0.75,0.90)
[0,0.75)
D
ICC
1.00
0.75
0.50
0.25
0.00
0
500
1000
Feature
Raw images
Processed images

Figure S3. Correlation analysis of volume-independent common prognostic RFs. The correlation coefficient matrices of EC (A) and NPC (B) showed that part features were highly correlated with absolute values of correlation coefficients greater than 0.6.

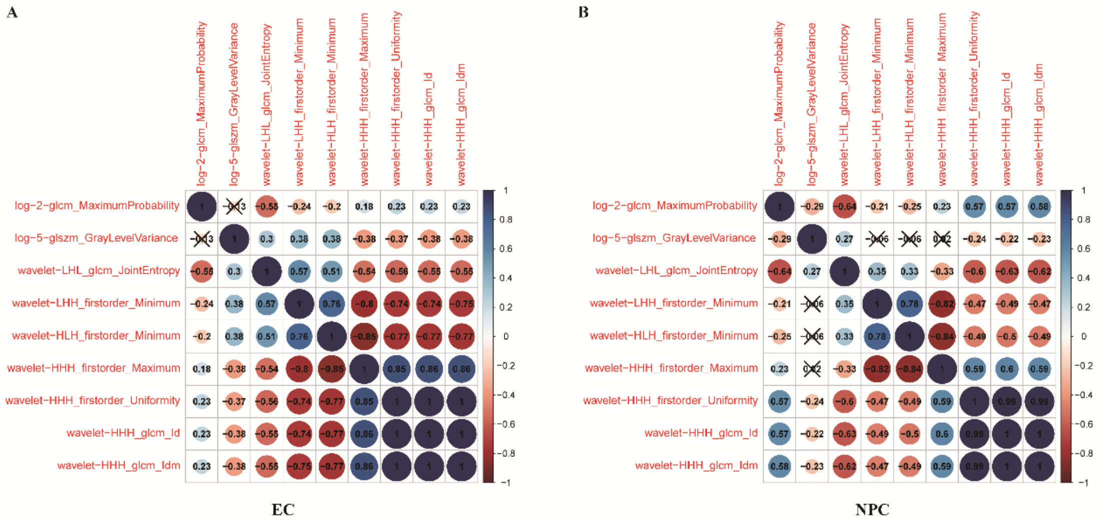

Figure S4. Comparison of prognostic performance between high- and low-repeatable RF groups. The prognostic performance of features from high-repeatable RF group and low-repeatable group was compared in training (A) and testing set (B). The RF generalization was evaluated using the absolute value of the difference between C-index of training and that of testing (|△C-index|) (C).

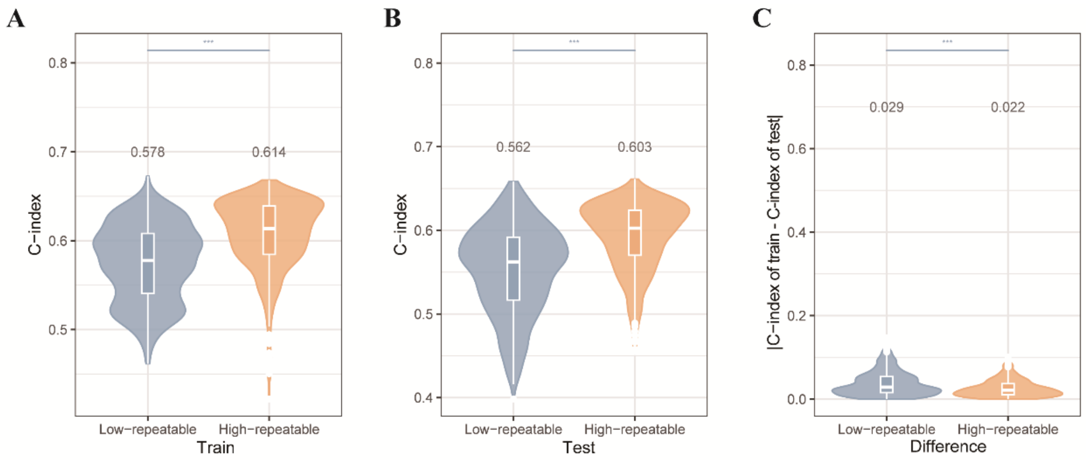

Figure S5. Kaplan-Meier survival analysis of the independent prognostic feature in the external testing set of EC. The LRFS of the two groups divided by original_shape_MeshVolume (A) and wavelet-HLH_firstorder_Minimum (D) showed significant difference, while the LRFS of the two groups divided by log-5mm-glszm_GrayLevelVariance (B) and wavelet-LHL_glcm_JointEntropy (C) was similar.

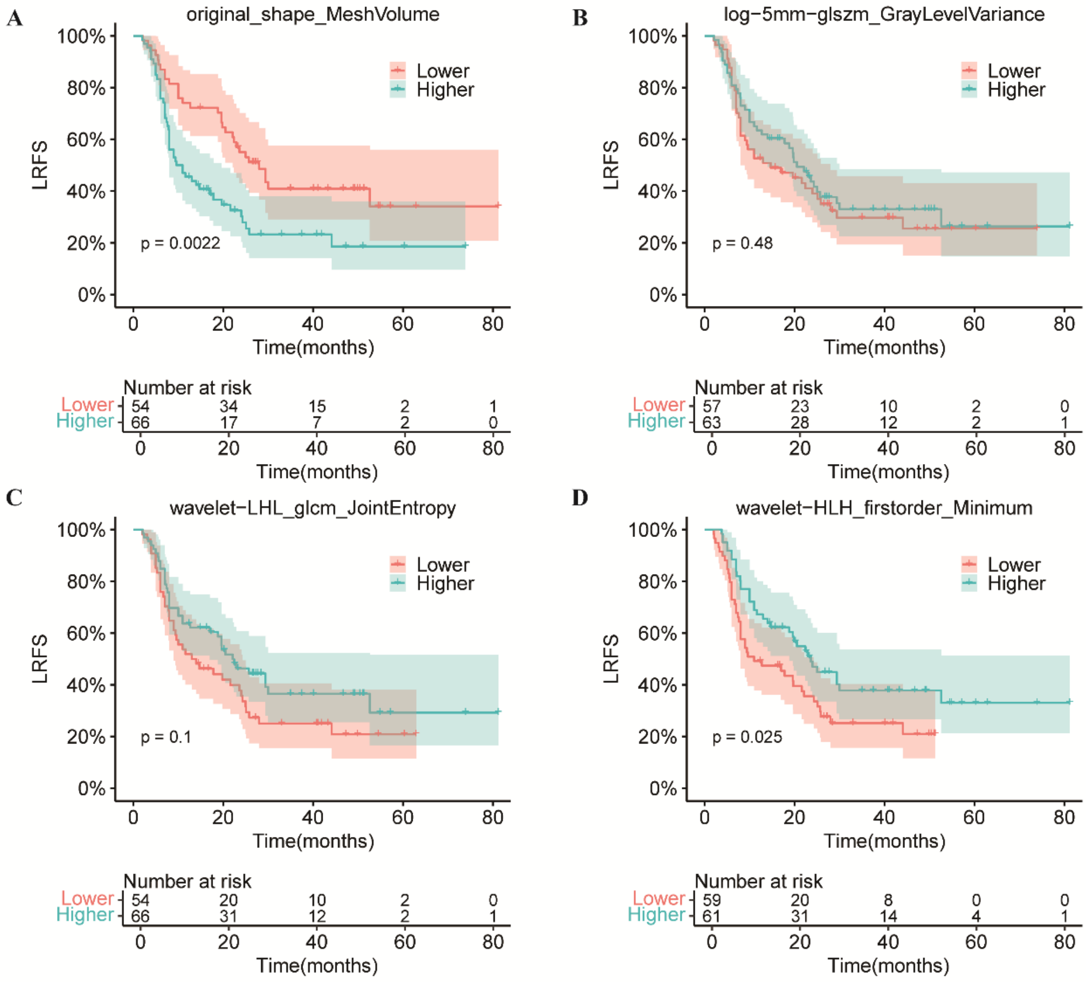

Supplement: Supplementary file 1 — ELECTRONIC SUPPLEMENTARY MATERIAL [file 13244_2025_2044_MOESM1_ESM.pdf]
